# Risk factors for postoperative delirium in patients with triple-branched stent graft implantation

**DOI:** 10.1186/s13019-020-01217-9

**Published:** 2020-07-14

**Authors:** Yanjuan Lin, Qiong Chen, Haoruo Zhang, Liang-Wan Chen, Yanchun Peng, Xizhen Huang, Yiping Chen, Sailan Li, Lingyu Lin

**Affiliations:** 1 Department of Nursing, Union Hospital, Fujian Medical University, No.29 Xinquan Road, Fuzhou, 350001 Fujian Province China; 2 Department of Nursing, Fujian Medical University, Fuzhou, China; 3 Department of clinical medicine, Fujian Medical University, Fuzhou, China; 4 Department of Cardiac Surgery, Union Hospital, Fujian Medical University, Fuzhou, 350001 Fujian China

**Keywords:** Risk factors, Postoperative delirium, Aortic dissection, Triple-branched stent graft implantation

## Abstract

**Background:**

Neurological complications is a common complication following novel triple-branched stent graft implantation in patients with Stanford type A aortic dissection (AAD). But the incidence and risk factors of postoperative delirium (POD) are not completely clear. The aim of this study was to investigate the incidence and risk factors of POD after novel triple-branched stent graft implantation.

**Methods:**

An observational study of AAD patients who underwent novel triple-branched stent graft implantation between January 2017 and July 2019 were followed up after surgery. Patients’ delirium was screened by the Richmond Agitation-Sedation Scale and the Confusion Assessment Method for the intensive care unit from the first day after the operation, lasted 5 days. The risk factors of POD were analyzed by the Cox proportional hazard models.

**Results:**

A total of 280 AAD patients were enrolled in this research, the incidence of POD was 37.86%. Adjusting for age, body mass index, and mechanical ventilation duration, multivariate Cox regression analysis model revealed that non-manual work (adjusted hazard ratio [AHR] = .554; 95% CI: 0.335–0.915; *P* = .021), Acute Physiology and Chronic Health Evaluation II (APACHE-II) scores > 20 (AHR = 3.359, 95% CI: 1.707–6.609, *P* < .001), hypoxemia (AHR = 1.846, 95% CI: 1.118–3.048, *P* = .017), and more than two types of analgesics and sedatives were independently associated with POD.

**Conclusions:**

This study showed that risk factors independently associated with POD were APACHE-II score > 20, hypoxemia, and more types of analgesics and sedatives, and non-manual work was the protective factor.

**Trial registration:**

This study was retrospectively registered in the Chinese Clinical Trial Registry (Registration number: ChiCTR1900022408; Date: 2019/4/10).

## Background

Aortic dissection (AD) is defined as an arterial disease in which the blood is separated from the aortic wall by the intimal tear so that the aortic lumen presents a pathological state of true and false lumen. Relevant studies have shown that the incidence of aortic dissection (AD) is 1–3/10,000 per year [1, 2]. According to the Stanford classification system, Stanford type A aortic dissection (AAD) is the most dangerous type of dissection which is involving the ascending aorta. As we know, surgery is the most effective treatment for AAD. In recent years, 90% of AAD patients have been treated by surgery [3]. Although the mortality rate after AAD has decreased significantly, the incidence of postoperative complications is still high, especially nerves systemic complications.

Postoperative delirium (POD) is a common neurological complication after heart surgery with a high incidence of 23.5–54.9% [4, 5]. POD is characterized by changes in arousal level and cognitive dysfunction, which is an acute and fluctuating cognitive dysfunction after anesthesia and surgery. Several studies have shown that patients with delirium lasting 1 day have a 20% increased risk of long-term hospitalization, and ones with delirium lasting 6 months to 1 year have a 10% increased risk of death [6]. In addition, delirium is closely related to decreased mobility, prolonged intensive care unit (ICU) stay, increased costs, and adverse clinical outcomes [7–9].

In recent years, some studies investigated the incidence of POD in patients with AD after Sun’s procedure, which was 32.5–52.0% [10, 11]. Their studies found that surgery duration, cardiopulmonary bypass (CPB) time, and selective cerebral perfusion (SCP) time were risk factors for delirium after AD. Although the novel triple-branched stent graft implantation significantly shortens the operation time, CPB time and aortic cross-clamping time by reducing vascular anastomosis of the branches of the aortic arch, nervous system complications remain one of the commonest and worst ones [12, 13]. The incidence and risk factors of delirium after the novel triple-branched stent graft implantation have not been studied.

Therefore, we carried out an observational study to assess the incidence and risk factors of delirium after novel triple-branched stent graft implantation in patients with AAD and to provide a scientific basis for formulating measures to reduce delirium in patients with AAD surgery.

## Methods

### Patients

An observational study of AAD patients who underwent novel triple-branched stent graft implantation between January 2017 and July 2019 was followed up after surgery. Inclusion criteria: 1) aged 18–75 years old; 2) patients and their families informed consent and volunteered to participate in this study. Exclusion criteria: 1) patients with the previous history of mental illness, stroke, and traumatic brain injury, or clinical or radiologic evidence of cerebral malperfusion by computed tomographic angiography; 2) congenital deaf-mute; 3) postoperative ICU stay < 24 h; 4) patients receiving continuous deep sedation with propofol and remifentanil after surgery. All patients were anesthetized and operated on by the same anesthesia group and the same treatment group. The endpoint of this study was delirium or not within 5 days after surgery. According to the results of the Confusion Assessment Method for the Intensive Care Unit (CAM-ICU), the subjects were divided into the delirium group and the non-delirium group. All participants or their families had informed consent and had the right to withdraw from this study at any time.

### Operative technique

Intravenous and inhalational combined anesthesia was applied in all patients. Anaesthetic medications used included sevoflurane, midazolam, sufentanil, rocuronium, and dexmedetomidine, which was administered according to the patient’s weight. The nasopharyngeal temperature, the rectal temperature, and upper and lower limb blood pressure were monitored. The Regional Oximetry System (VISTA, Covidien) was used to measure regional cerebral oxygen saturation. The depth of anesthesia was measured by the Bispectral Index (BIS) Monitoring System (VISTA, Covidien). The myocardial protection was performed by perfusion multiple administrations of cold blood cardioplegia (4 °C) through the left and right coronary arteries. Surgery was performed using combined low-flow CPB and cardiocirculatory arrest under deep hypothermia. The right axillary artery was used if it’s unilateral selective cerebral perfusion, and the right axillary artery and the left common carotid artery were used if it’s bilateral. The following procedures can be found in our previous literature [13].

### Delirium assessment

From the first day after surgery lasted until the fifth day after surgery, screening for delirium was performed daily through two registered nurses who had 3 years’ work experience in the cardiac surgery ward. Nurses were trained for 1 week by the complete Chinese version of the CAM-ICU training manual, to ensure the consistency of evaluation. The assessment of delirium consists of two steps. Step 1 was to assess patient’s level of consciousness by using the Richmond Agitation-Sedation Scale (RASS). If patient’s RASS score was − 4 or − 5, the patient is unconscious or in a coma, the assessment cannot continue. If patient’s RASS score range was − 3 ~ + 4, we moved on to step 2. Step 2 was to assess patient’s content of consciousness by using the CAM-ICU scale. This scale can identify the following four characteristics: include 4 features: 1) acute onset of change or fluctuation in mental status; 2) attention disorder; 3) altered level of consciousness; and 4) disorganized thinking. Delirium can be diagnosed if patient satisfies features 1, 2, 3, or 1, 2, and 4 at the same time.

### Data collection

This study collected thirty-five potential risk factors based on previous reports in the cardiovascular field [5, 14] (see Table 1 and Table 2). In this research, analgesics and sedatives included Morphine, Bucinnazine, Promethazine, Perphenazine, Chlorpromazine, Diazepam, etc. The non-manual worker in this study include teachers, government employees, doctors, editors, accountants, programmers, designers, and engineers. Manual worker mainly includes farmers, workers, and waiters (catering industry, sales industry). Ambiguous data needs to be checked again with the patients.

**Table 1:** Characteristics of delirium group and non-delirium group

Variables	Delirium (*N* = 106)	Non-delirium (*N* = 174)	*P*
Age, years, mean (SD)	57.15 (9.09)	49.47 (11.63)	< 0.001^a^
BMI (kg/m^2^), mean (SD)	25.64 (3.86)	24.38 (3.70)	0.007 ^a^
Male, n (%)	74 (69.8)	134 (77.0)	0.181^b^
Smoking, n (%)	40 (37.7)	74 (42.5)	0.428^b^
Drinking, n (%)	25 (23.6)	46 (26.4)	0.595^b^
Married	104 (98.1)	172 (98.9)	0.614^b^
Coronary heart disease, n (%)	2 (1.9)	3 (1.7)	0.921^b^
Hypertension, n (%)			0.01^b^
No	21 (19.8)	49 (28.2)	
Level 1	22 (20.8)	51 (29.3)	
Level 2	34 (32.1)	28 (16.1)	
Level 3	29 (27.4)	46 (26.4)	
Education level, n (%)			0.582^b^
Primary and below	57 (53.8)	83 (47.7)	
Middle school	32 (30.2)	62 (35.6)	
High school and above	17 (16.0)	29 (16.7)	
Nature of occupation, n (%)			0.001^b^
Manual worker	77 (72.6)	93 (53.4)	
Non-manual worker	29 (27.4)	81 (46.6)	
Medical payment method, n (%)			0.002^b^
At one’s own expense	41 (38.7)	38 (21.8)	
Medical insurance	65 (61.3)	136 (78.2)	
History of cardiac surgery, n (%)	6 (5.7)	5 (2.9)	0.253^b^
No	10 (94.3)	169 (97.1)	

^a^ Student’s t test. ^b^ χ^2^ test

*BMI* Body mass index

**Table 2:** Clinical data of delirium group and non-delirium group

Variables	Delirium (N = 106)	Non-delirium (N = 174)	*P*
**Preoperative data**			
First blood sugar (mmol/l), mean (SD)	8.54 (4.57)	7.16 (1.82)	< 0.001^a^
White blood cell (×10^9^/l), mean (SD)	11.27 (4.51)	11.63 (4.63)	0.518^a^
Neutrophil (× 10^9^/l), mean (SD)	11.38 (7.47)	12.42 (7.31)	0.250^a^
Red blood cell (×10^9^/l), mean (SD)	4.29 (0.79)	4.42 (0.604)	0.144^a^
Hemoglobin (g/l), mean (SD)	129.68 (20.83)	134.14 (18.09)	0.060^a^
Anemia, n (%)	15 (14.2)	24 (13.8)	0.933^b^
**Intraoperative data**			
State of consciousness, n (%)			0.781^b^
Quiet	43 (40.6)	78 (44.8)	
Irritability	45 (42.5)	68 (39.1)	
Coma	18 (17.0)	28 (16.1)	
ASA class, n (%)			0.304^b^
Level III	20 (18.9)	40 (23.0)	
Level IV and above	86 (81.1)	134 (77.0)	
Blood loss (ml), mean (SD)	842.26 (343.15)	809.20 (365.68)	0.453^a^
Allogeneic blood transfusion (ml), mean (SD)	359.06 (280.19)	334.20 (275.17)	0.467^a^
Erythrocyte transfusion, (u) mean (SD)	2.99 (2.88)	2.93 (2.71)	0.870^a^
Platelet transfusion, (u), mean (SD)	2.39 (2.82)	1.97 (2.94)	0.246^a^
Surgery duration (min), median (IQR)	288.5 (250.8334.3)	285.0 (249.5333.0)	0.877^c^
CPB (min), median (IQR)	142.0 (125.8171.3)	140.0 (115.0,176.0)	0.375^c^
Aortic cross-clamping duration (min), median (IQR)	60.0 (45.8,84.0)	52.0 (40.0,77.5)	0.010^c^
Lowest temperature, (°C) mean (SD)	23.22 (2.53)	23.37 (2.51)	0.629^a^
SCP (min), median (IQR)	12.0 (10.0,15.5)	12.0 (10.0,15.3)	0.168^c^
**Postoperative data**			
APACHE-II, mean (SD)	16.17 (4.05)	12.40 (3.33)	< 0.001^a^
Analgesics and sedatives, n (%)			< 0.001^b^
0–2	30 (28.3)	101 (58.0)	
3–4	63 (59.4)	63 (36.2)	
> 4	13 (12.3)	10 (5.7)	
Mechanical ventilation duration (hours), median (IQR)	63.5 (38.00,139.0)	40.32 (26.58,82.25)	< 0.001^c^
Hypoxemia, n (%)	41 (38.7)	27 (15.5)	< 0.001^b^
No	65 (61.3)	147 (84.5)	
ICU stay, median (IQR)	7.0 (5.0,9.0)	5.0 (4.0,7.0)	< 0.001^c^
Hospitalization days, median (IQR)	20.0 (15.0,26.0)	18.0 (14.0,23.0)	0.759^c^

^a^ Student’s t test. ^b^ χ^2^ test. ^c^ Wilcoxon rank-sum test

*ASA* American Society of Anesthesiologists, *SCP* Selective cerebral perfusion, *APACHE-II* Acute physiology and chronic health evaluation II, *ICU* Intensive care unit

Patients were divided into the delirium group and the non-delirium group according to whether delirium occurred or not. Sociodemographic and clinical categorical variables of two groups were evaluated using number and percentage values (%). Normality distributed numerical variables were described with mean and standard deviation (SD); medium and quartile range (QR) was used when it did no satisfied normal distribution. For normally distributed data, two groups were compared using Student’s t-test. For the comparison of non-normally distributed data, the Wilcoxon test was used. The Cox proportional hazard regression model: the number of days patients stay in ICU was taken as the time index, and the time of patients stay in ICU on the first day was taken as the starting point, calculated in units of days, and the truncated value was processed until patients developed delirium. Univariate and multivariate Cox regression analysis was performed with the occurrence of delirium as the dependent variable (event), and the hazard ratio (HR) value and 95% confidence intervals (CI) were obtained. In this study, Social science statistical software package (version 17.0) was used for all data’s statistical analysis. *P <* .05 was considered statistically significant.

## Results

**Fig. 1:**
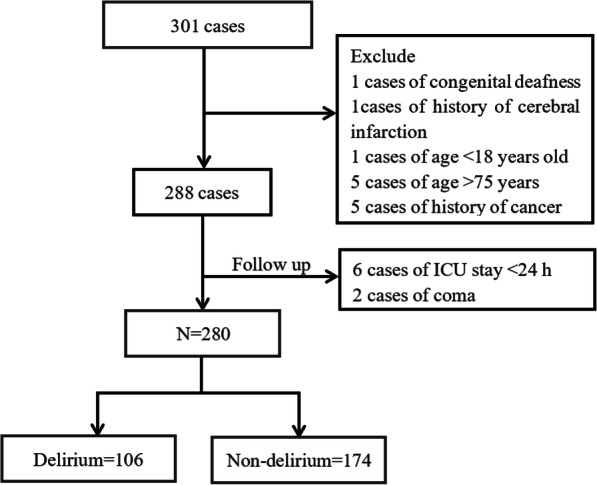
Patients’ flow chart

**Fig. 2:**
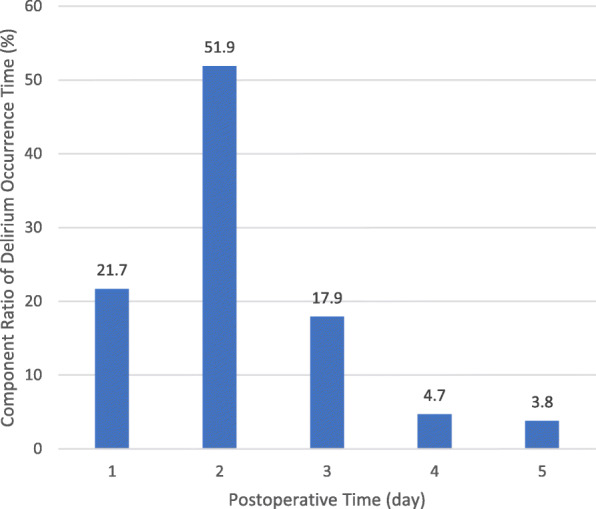
Composition ratio of POD in different periods of patients

From January 2017 to July 2019, 301 cases were admitted to the study, and the incidence of POD was 37.86% (The patient flow chart is shown in Fig. 1). The highest incidence of postoperative delirium was the second day after surgery (Fig. 2).

Table 1 shows the general data of two groups. There were significant differences in age, body mass index (BMI), hypertension, nature of occupational, medical payment, between the delirium group and the non-delirium group (*P <* .05).

As shown in Table 2. There were significant differences in blood sugar at the first admission, aortic cross-clamping time, Acute Physiology and Chronic Health Evaluation II (APACHE-II), analgesics and sedatives, mechanical ventilation duration, ICU stay, and hypoxemia between two groups (*P* < .05). There were no significant differences in other variables between the two groups (*P* > .05).

As shown in Table 3, age, BMI, hypertension grade, medical payment method, first blood sugar at admission, and mechanical ventilation duration were not correlated with the risk of POD (*P* > 0.05). Compared with the manual workers, non-manual workers can reduce the risk of POD (HR = .494; 95%CI: 0.308–0.792; *P* = .003). The HR of APACHE-II score ≤ 15–20 was 1.943 (*P* = .004), and APACHE-II score > 20 was 5.064 (*P* < .001). The use of more than two types of analgesics and sedatives increases the risk of POD (HR = 2.445; *P* < .05).

**Table 3:** Univariate Cox regression analysis results

Variables	β	SE	Wald	HR	95%CI	*P*
Age	0.006	0.011	0.308	1.006	0.985–1.027	0.579
BMI	0.002	0.027	0.952	1.002	0.949–1.057	0.952
Hypertension						
No	**Reference**					
Level 1	0.242	0.322	0.566	1.274	0.678–2.393	0.452
Level 2	0.619	0.297	3.342	1.857	1.037–3.325	0.068
Level 3	0.553	0.303	3.343	1.739	0.961–3.147	0.068
Nature of occupation						
Manual worker	**Reference**					
Non-manual worker	−0.706	0.241	8.582	0.494	0.308–0.792	0.003
Medical payment method	0.159	0.202	0.618	1.172	0.789–1.740	0.432
First blood sugar	0.009	0.021	0.168	1.009	0.967–1.052	0.682
Mechanical ventilation duration	0.001	0.001	0.269	1.001	0.998–1.003	0.604
APACHE-II score						
< 15	**Reference**					
≤15–20	0.664	0.233	8.126	1.943	1.231–3.069	0.004
> 20	1.622	0.303	28.740	5.064	2.779–9.165	< 0.001
ICU stay	−0.044	0.024	3.335	0.956	0.912–1.003	0.068
Hypoxemia	0.894	0.213	17.545	2.445	1.609–3.715	< 0.001
Analgesics and sedatives						
0–2	**Reference**					
3–4	0.896	0.266	11.381	2.450	1.456–4.123	0.001
> 4	1.760	0.387	20.650	5.811	2.720–12.413	< 0.001
Aortic cross-clamping duration	0.001	0.002	0.315	1.001	0.997–1.006	0.575

*SE* Standard error, *HR* Hazard ratio, *CI* Confidence intervals

*BMI* Body mass index, *APACHE-II* Acute physiology and chronic health evaluation II

As shown in Table 4, after adjusting for age, BMI, aortic cross-clamping time, and mechanical ventilation duration, a multivariable Cox regression analysis model showed that the risk of POD in non-manual workers was lower than that in manual workers (adjusted hazard ratio [AHR] = .554; *P* = .021). The patient APACHE-II score ≤ 15–20 was not associated with the risk of POD (AHR = 1.272; *P* = .362), and APACHE-II score > 20 (AHR = 3.359; *P* < .001) increased the risk of POD. The AHR of the types of analgesics or sedatives 3–4 was 2.122 (*P* = .016), and the AHR of the types of analgesics or sedatives > 4 was 3.383 (*P* = .005). Compared with that without hypoxemia, patients with hypoxemia are at increased risk of delirium (AHR = 1.846; *P* = .017).

**Table 4:** Multivariable Cox regression analysis results

Variables	β	SE	Wald	AHR	95%CI	*P*
Nature of occupation						
Manual worker	**Reference**					
Non-manual worker	−0.591	0.257	5.313	0.554	0.335–0.915	0.021
APACHE-II score						
< 15	**Reference**					
15–20	0.241	0.265	0.829	1.272	0.758–2.137	0.362
> 20	1.212	0.345	12.316	3.359	1.707–6.609	< 0.001
Hypoxemia	0.613	0.256	5.745	1.846	1.118–3.048	0.017
Analgesics and sedatives						
≤2	**Reference**					
3–4	0.753	0.311	5.855	2.122	1.154–3.904	0.016
> 4	1.219	0.437	7.777	3.383	1.436–7.967	0.005

*SE* Standard error, *AHR* Adjusted hazard ratio, *CI* Confidence intervals

Age, body mass index and mechanical ventilation duration have been adjusted

*APACHE-II* Acute physiology and chronic health evaluation II

## Discussion

This study aimed to analyze the incidence and risk factors of POD in patients with AAD who underwent novel triple-branched stent graft implantation. A total of 280 cases of AAD patients were enrolled in this research. There were 106 patients were diagnosed with delirium among them, and the incidence of delirium was 37.86%. In this research, we found that APACHE-II score > 20, types of analgesics and sedatives, and hypoxemia were independent risk factors for POD, and the non-manual worker was a protective factor for POD. At the same time, we did not find that aortic cross-clamping time and CPB time affected POD after AAD.

In the past 10 years, many studies have investigated the relationship between work and cognitive complexity. Marengoni et al. [15] and Smyth et al. [16] have shown that people who engage in physical work are more likely to have a risk of cognitive decline and dementia. Grzywacz et al. [17] found that work complexity was positively correlated with the cognitive function of episodic memory, executive function, and self-evaluation ability. The results of this research revealed that the non-manual workers in the delirium group after AAD were significantly less than those in the non-delirium group, and the results of multivariate Cox regression analysis showed that compared with the manual workers, the risk of delirium after the non-manual workers (AHR = .554; *P* = .015) was reduced. A variety of theories suggest that the nature of work may affect the cognitive function of the brain, among which the recognized cognitive reserve hypothesis indicates that the cognitive stimulation can increase the capacity of neurons, thus promoting the development of cognitive reserve [18]. For those who have been engaged in non-manual work for a long time, environmental stimulation may increase the level of neurotrophins available in brain tissue, protect or repair existing neurons, promote the development of nerves, and the incidence of neurological diseases will be relatively low [18].

Sedative and analgesic drugs work extensively on the central nervous system, including the nerve cell membrane, neurotransmitters, cerebral metabolism, and so on [19]. The main function of the central muscarine cholinergic system is to maintain cognitive function, while a variety of sedative and analgesic drugs act on the central alkaloid receptor [20]. Our study shows that the more analgesic and sedative drugs, the higher the risk of postoperative delirium. Related researches have indicated that the use of analgesics and sedatives is an independent risk factor for delirium after heart surgery. Pandharipande et al. [21] discovered that there was a positive correlation between benzodiazepines and the occurrence of delirium, and the incidence of delirium increased by 20% for every 1 mg increase of benzodiazepines. At the same time, the latest DAS-2015 recommendation [22] suggested avoiding benzodiazepines as far as possible, because benzodiazepines are easy to induce deep sedation, induced amnesia, and other side effects. Besides, commonly used opioid analgesics such as morphine can antagonize M1, M2, and M3 receptors at clinical doses [20]. In summary, the types of sedative and analgesic drugs should be reasonably selected according to the patient’s condition in clinical work.

According to reports, the incidence of hypoxemia after AD is 20.4–49.5% [23]. Kazmierski et al. [23] indicated that postoperative hypoxemia (PaO2 < 60 mmHg) was an independent risk factor for delirium after cardiac surgery. In this research, we found that the incidence of hypoxemia during and after AD surgery was 24.29%. Multivariate Cox regression analysis showed that hypoxemia increased the risk of delirium (AHR, 1.846; *P* < .001). AAD patients have accumulated blood and fluid in their chest cavity, and most of them need to undergo surgery under hypothermic circulatory arrest. Therefore, patients are prone to acute lung injury after surgery. Acute lung injury can lead to increased permeability of pulmonary vascular bed, pulmonary interstitial and alveolar edema, alveolar collapse, severe imbalance of ventilation/blood flow ratio, and eventually severe hypoxemia [24]. At the same time, hypoxemia can inhibit the synthesis of acetyl coenzyme A, acetylcholine, and glutamic acid in the citric acid cycle, resulting in decreased brain activity of cholinergic, which ultimately leads to an increased risk of delirium [13]. In addition, studies have shown that hypoxemia and mechanical ventilation time are interrelated. The more severe hypoxemia is, the longer mechanical ventilation time will be, and the risk of POD is increased.

The longer the duration of CPB, the greater the possibility of brain injury [25]. Surface contact between blood and CPB instrument can induce SIRS, which may be involved in the occurrence of delirium after operation through C-reactive protein, IL-1, and IL-10. Liu et al. [11] found that surgery duration (OR = 3.21; *P* = .002) was risk factors for POD. They speculated that prolonged surgery can lead to wider use of anesthetic drugs, increased blood transfusion, or the disturbance of electrolytes or acid-base balance. CPB time (OR = 1.360; *P* = .028) was also associated with a higher risk of POD. Prolonged CPB increases the release of inflammatory mediators, which can cause cerebral vasoconstriction, thereby inhibiting cerebral blood flow and changing brain function. However, none of our studies found that operative time and CPB time had an effect on POD. Shi et al. [10] and Liu et al. [11] found that the surgical methods were all Sun’s surgery, and this study was a three-branch stent surgery. By comparison, the operation time of delirium group was 288.5 (250.8, 334.3) and that of the non-delirium group was 285.0 (249.5, 333.0), which was 200 min shorter than those of Shi et al. [10] and Liu et al. [11] and the CPB time was 40–100 min shorter.

Our study was a single-center observational study, and larger sample size and multicenter trials are needed to determine risk factors for delirium after AAD surgery. We only evaluated delirium in ICU patients without long-term follow-up. The effect of dexmedetomidine on delirium was not discussed in this study and will be further explored in future studies.

## Conclusions

Our results showed that APACHE-II score > 20 and hypoxemia increased the risk of POD, and the more types of sedatives and analgesics, the higher the risk of delirium. And the risk of POD is reduced in non-manual workers. In addition, we did not find that aortic occlusion time and extracorporeal circulation time had an effect on delirium after AAD surgery.

## Data Availability

All data generated or analyzed during this study are included in this published article [and its supplementary information files].

## Abbreviations

AD: Aortic dissection
AAD: Stanford type A aortic dissection
POD: Postoperative delirium
ICU: Intensive care unit
CPB: Cardiopulmonary bypass
SCP: Selective cerebral perfusion
CAM-ICU: Confusion Assessment Method for the Intensive Care Unit
BIS: Bispectral index
RASS: Richmond Agitation-Sedation Scale
SD: Standard deviation
QR: Quartile range
HR: Hazard ratio
CI: Confidence intervals
BMI: Body mass index
APACHE-II: Acute Physiology and Chronic Health Evaluation II
AHR: Adjusted hazard ratio
